# Duplex Coating Combing Vanadate-Intercalated Layered Double Hydroxide and Ce-Doped Sol–Gel Layers on Aluminum Alloy for Active Corrosion Protection

**DOI:** 10.3390/ma16020775

**Published:** 2023-01-12

**Authors:** Kai Wei, Xuejie Zhao, Zhe Zhang, Yujie Yuan, Wenquan Kong, You Zhang

**Affiliations:** 1 College of New Materials and Chemical Engineering, Beijing Institute of Petrochemical Technology, Beijing 102617, China; 2 Beijing Key Lab of Special Elastomeric Composite Materials, Beijing 102617, China

**Keywords:** layered double hydroxide, silane, films, inhibitor, corrosion resistance, aluminum alloy

## Abstract

In this study, a duplex coating system (LDH-V/SG-Ce) of vanadate-intercalated layered double hydroxide (LDH) and Ce-doped sol–gel (SG) layers was developed for the purpose of active corrosion protection of the aluminum alloy AA2024. ZnAl-LDH film was grown in situ on the surface of an aluminum alloy using a hydrothermal method and intercalated with V_2_O_7_^4−^ anions as corrosion inhibitors, and sealed with a Ce (III)-doped silane coating using a sol–gel technique. Scanning electron microscopy (SEM), Fourier transform infrared spectroscopy (FTIR) and X-ray diffraction (XRD) analyses were used to analyze the microstructure, surface functional groups and structure of the LDH-V/SG-Ce film. The uniform and compact silane layer was covered both in the pores and on top of the LDH film. The results of glow discharge optical emission spectroscopy (GDOES) indicated that V_2_O_7_^4−^ and Ce (III) ions were loaded in the LDH layer and silane film, respectively. The potentiodynamic polarization results showed that the corrosion current density of the bilayer system in the presence of corrosion inhibitors was reduced to 1.92 × 10^−8^ A/cm^2^. Electrochemical impedance spectroscopy (EIS) results showed that the LDH-V/SG-Ce duplex coating could provide effective protection for the aluminum alloy after being exposed to a corrosive solution for 14 days.

## 1. Introduction

As a structural material, aluminum alloy has a wide range of applications, especially in the fields of building, electrical and aerospace engineering [1,2,3,4]. Uncontrolled corrosion damage of metal is easily ignored by people, and often leads to mechanical failure or even disastrous accidents. In the past, chromate conversion coating was mainly used as one of the most effective methods in aluminum alloy surface treatment technology. However, Cr^6+^ can cause serious pollution to the environment and even serious harm to human health [5,6,7]. Research on the development of new anticorrosion coatings, such as anodic oxidation [8,9], electroplating [10,11], organic coatings [12,13] and LDH [14,15,16], is of great significance for replacing chromate conversion coatings and realizing the long-term corrosion protection of aluminum alloys. With a single film system, it is often difficult to meet the corrosion resistance requirements of practical application scenarios of aluminum alloys. The method of constructing composite films can effectively combine the advantages of multiple films and overcome the defects of a single film. To construct composite films, it is necessary to consider the functionality of each film and the adhesion between the films. At present, a film surface with good mechanical performance is usually used as the topcoat to resist the erosion of the external environment. When the outer film loses its function, the bottom film, coated with a corrosion inhibitor, will prevent corrosion of the solution by releasing the corrosion inhibitor [17]. The method of loading corrosion inhibitors directly determines whether the composite films can achieve long-term corrosion resistance.

Layered double hydroxide (LDH) can be used as a smart protective film for storing corrosion inhibitors due to its unique 3D nanosheet array layered structure and interlayer anion exchange capacity [18,19,20]. Using in situ growth, LDH film can form a system composed of interlayer anions and metal cations with good adhesion on the metal surface [21,22]. The intermediate layer can retard the corrosion process by absorbing the most important corrosive chloride ions. At the same time, the intermediate layer can also be used as a storage location for anionic corrosion inhibitors [23,24]. An inherently self-healing and antibacterial film of inorganic Li-Al layered double hydroxide film modified by adding 2-guanidinosuccinic acid (GSA) was prepared on the surface of a 6N01-T5 aluminum alloy, which has enhanced corrosion resistance. After the coating is damaged, the interlayer GSA is released and adsorbed on the metal substrate surface to form a film [25]. Various anionic corrosion inhibitors have been successfully intercalated in LDH films, and the anticorrosion performance of films loaded with different corrosion inhibitors on the AA2024 substrate has been compared. Among them, vanadate anion has the best performance in providing long-term anticorrosion effects [26]. LDH nanocontainers doped with vanadate anions as corrosion inhibitors offer a well-defined self-healing effect and confer corrosion protection properties by direct synthesis and anion-exchange approaches [27].

However, the surface of LDH film is rough, due to the nonuniformity of the LDH grain growth process. There are a lot of defects at the micrometer level where the corrosive solution can reach the substrate [28]. Using a sealing treatment with silane sol–gel coating can improve the defects of the LDH layer and the adhesion of silane film on the surface of an aluminum alloy. As the first gateway to resist corrosion, a silane sol–gel coating can also realize the loading and release of different corrosion inhibitors in multilayers through inhibitors (especially cationic inhibitors) [29].

A double-layer system consisting of an LDH conversion film covered by a sol–gel film containing vanadate inhibitor was used in a study of the active corrosion protection on AA2024. This system has efficient active corrosion protection and self-healing capability [30]. In our previous work [31], a double-doped LDH container containing cerium and vanadate ions was prepared using a two-step method. The results showed that the double-doped film provides enhanced corrosion resistance, and that there is a potential synergistic effect between Ce and V_2_O_7_^4−^.

In this study, a vanadate-intercalated LDH and cerium-doped sol–gel (SG) double coating system (LDH-V/SG-Ce) was developed for active corrosion protection of the aluminum alloy AA2024. To enhance the corrosion protection of AA2024, the potential synergistic effect between Ce and VO_3_^−^ in the dual inhibitor system was studied.

## 2. Materials and Methods

### 2.1. Preparation of Materials

The aluminum alloy AA2024 plates (with a nominal composition in wt.% of Cu 3.9, Mg 1.2, Mn 0.42, Cr 0.1, Zn 0.24, Ti 0.1 and balanced Al) used in this work were cut to 50 mm × 50 mm × 5 mm size for convenient processing. The samples were progressively polished to 1500 grit with SiC sandpapers, cleaned in deionized water and ethanol, and dried using cold air.

### 2.2. Synthesis of LDH-V Film by Anion-Exchange Reaction

A 300 mL mixed solution of 0.05 M Zn(NO_3_)_2_·6H_2_O (99 wt.%, Aladdin, Shanghai, China) and 0.3 M NaNO_3_ (AR, Sinopharm Chemical Reagent Co., Ltd., Shanghai, China) was prepared with pH in the neutral range (6.3) by adding 1% ammonia. The aluminum alloy samples were placed vertically in the mixed solution at a constant temperature of 70 °C for 24 h. After the reaction, the samples were taken out, rinsed with deionized water and ethanol (>98%), and then dried in cold air. After the above steps, LDH-N films were prepared on the surface of the samples.

LDH-V films were obtained by immersion of LDH-N samples in 0.1 M NaVO_3_ (AR, Macklin, Shanghai, China) solution (pH = 8.8). The anion-exchange reaction was carried out at 45 °C for 2 h. After immersion, the fabricated films were washed with deionized water and ethanol, then dried in cold air.

### 2.3. Synthesis of LDH-V/SG-Ce Film

As starting materials, 30 mL ethanol was dissolved in 14 mL deionized water. Then, 24 mL γ-glycidyloxypropyltrimethoxysilane (GPTMS, C_9_H_20_O_5_Si, 97%, Aladdin, Beijing, China) was slowly added to the obtained mixture. The mixed solution was stirred for 1 h to obtain gel A. For gel B, 6 mL ethyl acetoacetate (C_6_H_10_O_3_, 99.8%, Beijing Chemical Works, Beijing, China) was dissolved in 12 mL ethanol. Next, 12 mL tetraethylorthosilicate (TEOS, C_8_H_20_O_4_Si, 98%, Macklin, Shanghai, China) was slowly added into the mixed solution. To adjust the pH, 1.2 mL glacial acetic acid (CH_3_COOH, AR, Beijing Chemical Works, Beijing, China) was added. The resulting solution was stirred for 1 h to obtain gel B. The prepared gel B was dropped into gel A and stirred for 30 min. Next, 0.01 M Ce(NO_3_)_3_·6H_2_O (99.5%, Macklin, Beijing, China) was added into the solution, with stirring at 25 °C for 30 min to obtain the gel-doped Ce^3+^ we named SG-Ce. The previously prepared LDH-V sample was coated using a static dip-coating method at a pulling rate of 90 mm/min. After the lifting, the surfaces of the samples needed to be dried in cold air to prevent the gel from gathering at the hole and causing sagging. The samples were sequentially subjected to 60 °C for 3 h, 90 °C for 1 h, and 120 °C for 30 min using the temperature gradient drying method. Three parallel samples were required in order to study the morphology, composition, and corrosion performance.

### 2.4. Characterization

The crystal structure of LDH was measured with the scanning range from 3 to 70° using glancing angle X-ray diffraction (GAXRD) with Cu Kα1 (λ = 0.154 nm) radiation. The surface morphology of the synthesized LDH was detected using a field emission scanning electron microscope (SEM, JEOL JSM-7800) under 10 kV accelerating voltage, and energy dispersive spectrum (EDS, Oxford Instrument Isis 300) was used to study the chemical composition. All samples were sprayed with a gold layer to provide surface conductivity. The types of functional groups and chemical bonds in LDH were obtained from Fourier transform infrared (FTIR) spectra using a Bruker Tensor 27 OPUS spectrometer in the range of 4000–400 cm^−1^. The samples were quantitatively analyzed using glow discharge optical emission spectroscopy (GDOES, HORIBA GD Profiler 2). At 650 Pa pressure and 30 W power, the depth profile of the samples was analyzed using copper with a diameter of 4 mm as the anode. Electrochemical impedance spectroscopy (EIS) and potentiodynamic polarization measurements were carried out using the Wuhan Corrtest CS350 electrochemical workstation. A typical three-electrode system was used to measure the electrochemical behavior of the film. The sample to be tested was a working electrode with an exposure area of 1 cm^2^, a high-purity platinum plate as the counter electrode, and an Ag/AgCl electrode as a reference electrode. The tests were carried out in a cell using 3.5 wt.% NaCl solution at room temperature. After the open circuit potential (OCP) was stabilized, the electrochemical impedance spectroscopy (EIS) test was conducted. The amplitude of the sine wave was ±10 mV, and the frequency range was 10^4^–10^−2^ Hz. The impedance spectrum data obtained were simulated by an equivalent circuit. The scanning speed of the Tafel dynamic potential polarization test was 1 mV·s^−1^, and the scanning range was from −0.25 V to +0.5 V (relative to OCP).

## 3. Results

### 3.1. Microstructure and Chemical Composition of the Coatings

**Figure 1:**
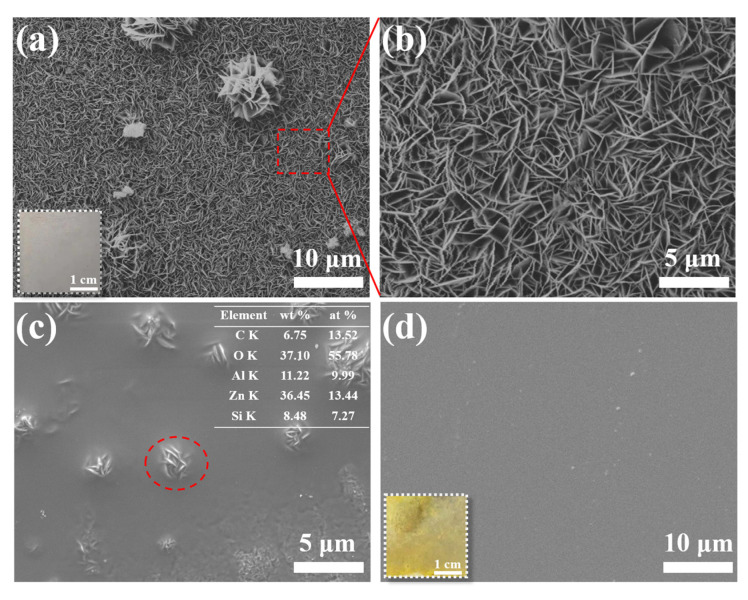
SEM images of (**a**,**b**) LDH-N film, (**c**) LDH-V/SG-Ce film (one layer of SG, red dotted circle for EDS detection) and (**d**) LDH-V/SG-Ce film (two layers of SG).

Figure 1a,b illustrate the microscopic morphologies of LDH-N film, showing the typical LDH film morphology. The LDH layer formed by the interconnection of hexagonal nanosheets about 2–3 μm in size grows compactly and completely and covers the surface of the AA2024 substrate. It is worth noting that several LDH clusters grow above the compact layer, which may be due to the influence of intermetallics of the AA2024 substrate on the growth process of LDH nanocrystals [32]. The LDH film on the AA2024 surface is basically completely covered after being dip-coated once using the sol–gel method. Except for the flower cluster positions, the LDH film is mainly entirely covered, as shown in Figure 1c. The presence of Si in the LDH flower clusters indicates that the sol–gel matrix would infiltrate into the LDH pores during the curing process. This approach can effectively improve the binding force between LDH and SG layers. However, uneven coverage at the top of LDH flower clusters may cause a lot of stress and cracks or other defects could form [33]. After dip-coating twice, as shown in Figure 1d, the LDH-V/SG-Ce composite film has good uniformity, and the surface is flat without obvious defects.

**Figure 2:**
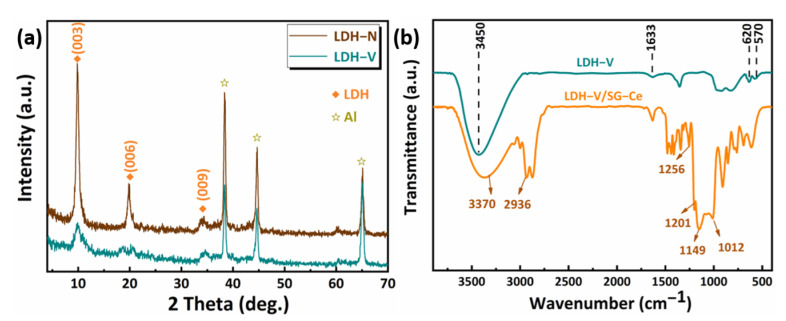
(**a**) XRD patterns of LDH-N and LDH-V films; (**b**) FTIR spectra of LDH-V and LDH-V/SG-Ce films.

The XRD patterns of LDH films on AA2024 substrates are depicted in Figure 2a. The main characteristic reflections (003) and (006), at about 10 and 20°, respectively, represent the formation of LDH intercalated with NO_3_^−^. After the anion-exchange reaction, the positions of the characteristic peaks are almost unchanged. The diffraction reflection (003) shifts to a lower angle, which indicates that the vanadate anion is successfully intercalated in the form of HV_2_O_7_^3−^/V_2_O_7_^4−^ [34]. Figure 2b shows the FTIR spectra of the LDH-V films before and after modification with gel-doped Ce^3+^. A strong absorption band around 3450 cm^−1^ can be identified as the hydroxyl stretching band *m* (OHstr), which is caused by the interlayer water molecules in the LDH nanosheet array structure and the water adsorbed on the surface of silane film. Another weak band centered at around 1633 cm^−1^ is generated by the hydroxyl deformation mode of water δ (H_2_O) [14]. In the range 500–800 cm^−1^, the bands are attributed to M-O and M-O-M lattice vibrations [26]. The bands around 620 and 570 cm^−1^ in the LDH-V film may come from the V-O-V bond, while the absorption peak around 964 cm^−1^ may come from the V-O bond [35]. Combined with XRD analysis, the successful intercalation of vanadate anion in LDH can be obtained. For the LDH-V/SG-Ce film, the peak at around 1256 cm^−1^ is attributed to the vibration of epoxy groups [36]. The absorption bands around 1201 cm^−1^ and 3370 cm^−1^ are associated with the Si-O-CH_3_ and Si-OH vibrations, respectively [37]. The C-H peak around 2936 cm^−1^ belongs to the carbon chain in silane [38]. Two characteristic peaks at 1149 cm^−1^ and 1012 cm^−1^ are attributed to the stretching vibration of the Si-O-Si bond in the silicon-based network structure in the film [39]. By comparing the infrared spectra of LDH-V and LDH-V/SG-Ce films, it was found that the characteristic absorption peaks of LDH almost disappeared. The results of FTIR and SEM analysis indicate that SG layers are successfully prepared and covered on the surface of LDH film.

**Figure 3:**
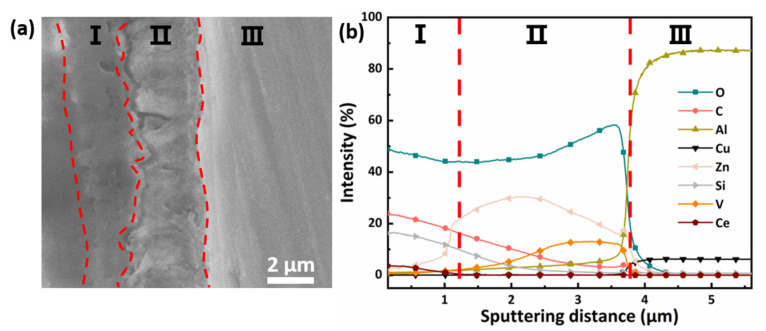
(**a**) Cross-section SEM image and (**b**) GDOES depth profile of LDH-V/SG-Ce (I: SG film; II: LDH film; III: AA2024 matrix).

Figure 3a shows a cross-section SEM image of LDH-V/SG-Ce film. Regions I, II, and III represent SG, LDH film, and the AA2024 matrix, respectively. The SG film of 1–2 μm covers the LDH film of 2–3 μm. It can be clearly seen that the SG and LDH layers are cross-linked at the interface [17]. The SG layer infiltrates into the LDH layer, indicating that they are tightly combined. In the GDOES diagram in Figure 3b, the element content of Si in Region II gradually decreases; this also proves that the SG matrix penetrates the LDH film. The element content of O is relatively high in Regions I and II, due to the main film-forming material, SiO_2_, in the SG layer and Al(OH)_4_^−^ and Zn(OH)^+^ in the LDH film, respectively [40]. The gradual decrease of Zn in Region I may come from LDH flower clusters being covered by SG coating. The element content change of Cu can be used as the basis to distinguish between LDH film and AA2024 substrate. The distribution of V in LDH film is close to the AA2024 substrate surface, which indicates that the intercalation of vanadate anion is successful. A small amount of Ce is detected, proving that it is feasibly loaded in the SG film [41].

### 3.2. Corrosion Behavior of the Coating

**Figure 4:**
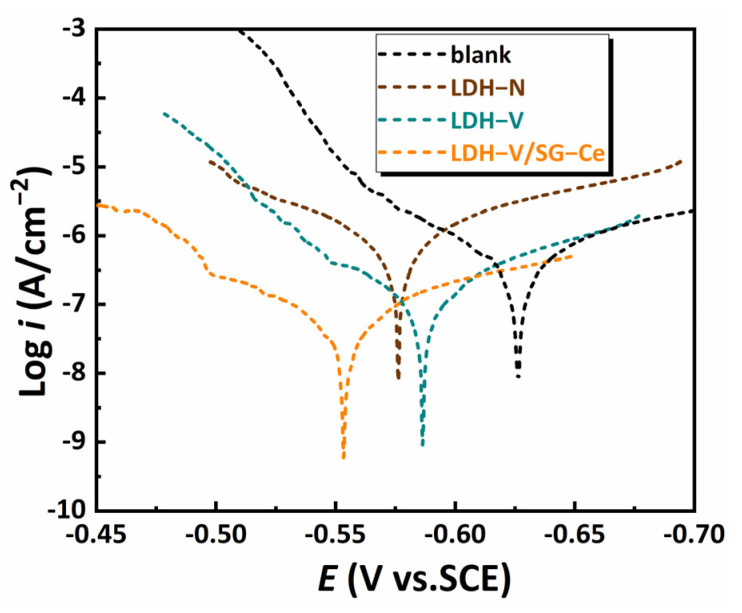
Potentiodynamic polarization curves for samples exposed in 3.5 wt.% NaCl solution.

Figure 4 shows the potentiodynamic polarization curves of different film systems exposed in 3.5 wt.% NaCl solution. Table 1 shows a series of parameters of potentiodynamic polarization curves obtained using the Tafel linear extrapolation method, including corrosion potential (*E_corr_*), corrosion current (*I_corr_*), anode slope (*b_a_*) and cathode slope (*b_c_*). The *I_corr_* of the LDH-V decreases, and the *E_corr_* moves to a negative direction, after the intercalation of the vanadate anion. Moreover, the value of *b_c_* becomes higher, indicating that the vanadate anion as a cathodic inhibitor inhibited the corrosion process on AA2024 [42]. The *E_corr_* of LDH-V/SG-Ce film added with double corrosion inhibitors increases significantly to −0.554 V, and the slope of the polarization curve in the anode area shifts, indicating that Ce^3+^ acts on the AA2024 surface in the form of an anodic corrosion inhibitor [43]. The *I_corr_* reaches the minimum value, which is nearly two orders of magnitude lower than that of the AA2024 matrix. This is due to the sealing effect of silane film on LDH film, which prevents the corrosion solution from penetrating the film. The products formed by two types of corrosion inhibitors on the surface of AA2024 prevent the corrosion solution from making contact with the metal surface. The synergistic effect of the Ce^3+^ anode type and vanadate anion cathode type inhibitors inhibited corrosion.

**Figure 5:**
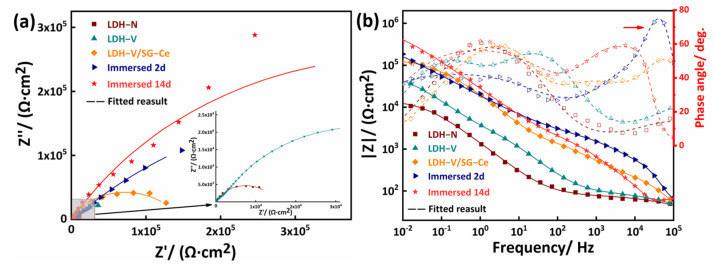
(**a**) Nyquist plots and (**b**) Bode plots of LDH and LDH/SG samples exposed in 3.5 wt.% NaCl solution for 2 days and 14 days (gray part in (**a**) is a partial enlargement).

EIS measurement was used to investigate the anticorrosion performance of the samples exposed in 3.5 wt.% NaCl solution. EIS data are shown as both Nyquist and Bode impedance plots, as presented in Figure 5a,b, respectively. Three relaxation time constants can be observed in the Bode plot of Figure 5b: (i) high-frequency range (10^3^–10^5^ Hz) is associated with the presence of sol–gel coating at the interface between coating and corrosive solution; (ii) intermediate-frequency range (10^0^–10^2^ Hz) is associated with the presence of SG/LDH layer; (iii) low-frequency range (10^−2^ Hz) is related to corrosion activity [28]. The capacitive arc radius of the LDH-V/SG-Ce is the largest, meaning it has the best corrosion resistance. The higher the impedance modulus in the low-frequency region, the better the corrosion resistance of the coating [19]. The highest impedance modulus at a low frequency of LDH-V/SG-Ce, which is nearly an order of magnitude higher than the LDH film, also indicates this point. This is due to the physical barrier effect formed by the good combination of the sol–gel film and the LDH film.

It is worth noting that the capacitive arc radius of the LDH-V/SG-Ce sample is significantly increased and the low-frequency impedance modulus is stable at taller than 10^5^ Ω·cm^2^ after immersion in 3.5 wt.% NaCl solution for 2 and 14 days, indicating that corrosion resistance becomes stronger with the extension of immersion time. This is related to the release of corrosion inhibitors of Ce^3+^ and vanadate anion during immersion. At the early stage of immersion (2 days), vanadate ions are released and adsorbed on the surface of the substrate at the most corrosion-prone alloy phase position [44]. With extended immersion time, Ce ions reach the substrate surface with the corrosive solution and form a Ce oxide film to prevent the corrosive solution from corroding the substrate [45].

**Figure 6:**
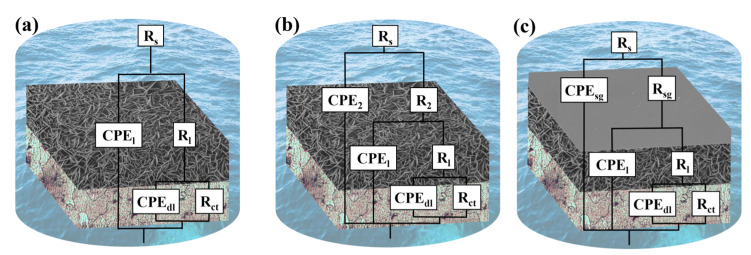
Equivalent circuits employed for fitting the impedance spectra of LDH-N coated system (**a**), LDH-V coated system (**b**) and LDH-V/SG-Ce coated system (**c**).

The electrical equivalent circuits (EEC) shown in Figure 6 are used to fit the EIS results. Figure 6a–c show the EEC of LDH-N, LDH-V and LDH-V/SG-Ce samples, respectively. Table 2 lists the key parameters for EEC calculation. R_s_, R_sg_, R_ct_, R_1_ and R_2_ represent solution resistance, silane film resistance, adsorption film of vanadate anion resistance, oxide of LDH film resistance and charge transfer resistance, respectively [46,47]. CPE_sg_, CPE_2_, CPE_1_ and CPE_dl_ represent SG film capacitance, adsorption film capacitance, LDH film capacitance and electric double layer capacitance, respectively. Obviously, the highest R_ct_ and lowest CPE_dl_ indicate that the LDH-V/SG-Ce sample has excellent corrosion resistance.

## 4. Discussion

**Figure 7:**
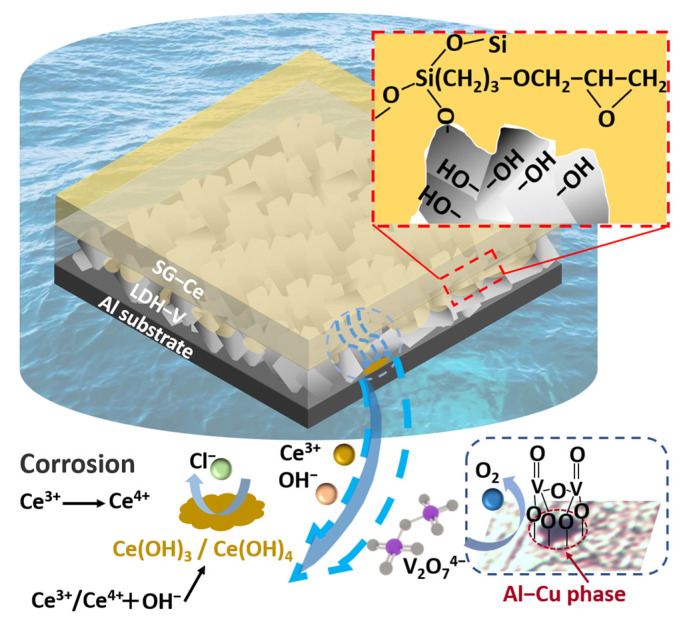
Schematic representation of coating formation mechanism of LDH/SG composite film.

As described in Figure 7, the corrosion resistance mechanism of the composite coating can be divided into the following three points: (i) After the hydrolysis of the sol–gel film, a large number of silane alcohol groups are generated. These groups combine with the metal hydroxide of the LDH film during the curing process to form covalent bonds of Si-O-Si and Si-O-Me, making the sol–gel layer closely combine with the LDH film [48]. (ii) In the corrosion process of an aluminum alloy, aluminum spontaneously oxidizes to form an oxide film, which creates a local weak acidic environment. The aluminum alloy AA2024 contains a variety of alloying elements, resulting in an uneven microstructure. It is composed of multiple intermetallic phases (IMPs), such as CuAl_2_ and ZnAl_2_. In addition, these areas lack protective oxide film and are prone to local corrosion and intergranular corrosion. Vanadate anion, in the form of V_2_O_7_^4−^, inhibits the adsorption of oxygen at these sites, thus inhibiting the redox reaction and hindering corrosion with the cathodic corrosion inhibitor [42,47,49]. (iii) During the corrosion process, Ce^3+^ is partially oxidized to Ce^4+^ and attaches to the aluminum alloy substrate in the form of Ce_2_O_3_/Ce(OH)_3_ or CeO_2_/Ce(OH)_4_ to prevent further corrosion [45,50,51,52]. Eventually, the synergistic effect of the two inhibitors together inhibits the occurrence of corrosion.

## 5. Conclusions

In summary, the duplex coating system (LDH-V/SG-Ce) consisting of vanadate-intercalated layered double hydroxide (LDH) and cerium-doped sol–gel (SG) system has been demonstrated to be useful for the active anticorrosion of an aluminum alloy. The ZnAl-LDH film was grown in situ on an aluminum alloy surface using a hydrothermal method, intercalated with V_2_O_7_^4−^ anions as corrosion inhibitors, and sealed with a Ce (III)-doped silane coating using a sol–gel technique. Microscopic morphology and composition tests revealed the LDH layer to have many micropore defects, and flower cluster protrusions on the surface were completely coated by a SG-Ce film. On the other hand, the penetration of the sol–gel layer into the LDH layer greatly enhances the binding force. GDOES tests showed that the thickness of the composite coating was 3–4 μm and that two inhibitors were successfully loaded. EIS measurements showed that the impedance modulus of the LDH/SG coating increased by one order of magnitude after being exposed to a 3.5 wt.% NaCl solution for 14 days, indicating that the release of the corrosion inhibitor forms a new film on the surface of the substrate, and effectively improves the composite corrosion resistance. The polarization curve indicates that the corrosion protection mechanism of AA2024 by composite coating may come from the synergistic effect of the anode corrosion inhibitor (Ce) and cathode corrosion inhibitor (V_2_O_7_^4−^). Furthermore, the corrosion current density of the bi-layer system was reduced to 1.92 × 10^−8^ A/cm^2^ in the presence of corrosion inhibitors. The composite coating system effectively implemented the function of active corrosion protection, and provided a new idea for a multi-layer corrosion protection system.

## Figures and Tables

**Table 1:** Parameters of potentiodynamic polarization curves for samples exposed in 3.5 wt.% NaCl solution.

Title 1	*E_corr_* (V)	*I_corr_* (A/cm^2^)	*b*_a_/(V/dec)	*b*_c_/(V/dec)
Blank	−0.624	1.457 × 10^−6^	0.246	0.324
LDH-N	−0.576	3.570 × 10^−7^	0.263	0.279
LDH-V	−0.587	0.795 × 10^−7^	0.227	0.235
LDH-V/SG-Ce	−0.554	1.920 × 10^−8^	0.342	0.348

**Table 2:** EIS fitting parameters of the coating systems.

Sample	LDH-N	LDH-V	LDH-V/SG-Ce	Immersing 2 Days	Immersing 14 Days
R_s_/Ω cm^2^	26.42	7.34	28.18	60.77	67.76
CPE_sg_/μF cm^−2^	/	/	3.63 × 10^−8^	4.83 × 10^−10^	6.63 × 10^−10^
R_sg_/Ω cm^2^	/	/	166.10	355.01	84.59
CPE_2_/μF cm^−2^	/	2.89 × 10^−5^	/	/	/
R_2_/Ω cm^2^	/	5125	/	/	/
CPE_l_/μF cm^−2^	1.92 × 10^−5^	1.68 × 10^−5^	1.05 × 10^−5^	7.19 × 10^−7^	1.26 × 10^−6^
R_l_/Ω cm^2^	53.86	92.01	2666	1743	1851
CPE_dl_/μF cm^−2^	1.70 × 10^−4^	5.63 × 10^−5^	3.43 × 10^−6^	2.14 × 10^−5^	9.46 × 10^−6^
R_ct_/Ω cm^2^	14,951	69,257	146,150	853,230	889,010

## Data Availability

Not applicable.
